# VSELs Maintain their Pluripotency and Competence to Differentiate after Enhanced Ex Vivo Expansion

**DOI:** 10.1007/s12015-018-9821-1

**Published:** 2018-05-08

**Authors:** Rachid Lahlil, Maurice Scrofani, Romain Barbet, Céline Tancredi, Anne Aries, Philippe Hénon

**Affiliations:** Hôpital du Hasenrain, Institut de Recherche en Hématologie et Transplantation (IRHT), 87 avenue d’Altkirch, 68100 Mulhouse, France

**Keywords:** VSELs, Human very small embryonic-like stem cells, Umbilical cord blood, UM171, CD34+/CD133+/CXCR4+ cells, NANOG

## Abstract

The very small embryonic-like stem cells (VSELs) are known as a subset of adult pluripotent stem cells able to differentiate to all three germ layers. However, their small number and quiescence restrict the possibility of their use in cell therapy. In the present study, we first delineate different subpopulation of VSELs from human cord blood CD34+ cells to define their purity. We next determine genes expression levels in the whole transcriptome of VSELs expressing the pluripotent marker NANOG and control cells under the steady state condition. We found that more than a thousand of genes are downregulated in VSELs, as well as many membrane receptors, cells signaling molecules and CDKs mRNAs. In addition, we observed discordance in some pluripotent genes expression levels with embryonic stem cells (ESCs), which could explain VSELs quiescence. We then evaluate VSELs capacity to expand and differentiate in vitro in specific and appropriate media. After 12 days culture in specific medium containing a pyrimidoindole derivative (UM171), VSELs were significantly expanded for the first time without feeder cells and importantly preserve their capacities to differentiate into hematopoietic and endothelial cells. Interestingly, this stimulation of VSELs self-renewal restores the expression of some downregulated genes known as key regulators of cell proliferation and differentiation. The properties of such pluripotent expanded cells make them a potential candidate in regenerative medicine.

**Electronic supplementary material:**

The online version of this article (10.1007/s12015-018-9821-1) contains supplementary material, which is available to authorized users.

## Introduction

Very small embryonic-like stem cells (VSELs) are present in the bone marrow, peripheral blood as well as in umbilical cord blood (UCB), and can give rise to cells from all three germ layers [1–7]. However, compared with other source, UCB has become an attractive source of stem cells including VSELs with a large accessibility and tolerance to allogenic graft, but still limited by a low number of stem cells countenance [8]. VSELs can be isolated on the basis of phenotypic features like their small size (2 to 6 μm), are Lin-, CD34+, CD45-, CD133+ and/or CXCR4+ [7, 9, 10]. VSELs express several pluripotent genes such as Oct-4, NANOG, Klf-4 and SSEA-4 and primordial germ cells markers reviewed in [11]. They are actively mobilized from the bone marrow into peripheral blood following stressful conditions such as stroke [12, 13], myocardial infarction [14], critical leg ischemia [15], pulmonary diseases [16] or cytotoxic treatments [13]. Similar or overlapping populations of these pluripotent stem cells have been described following different experimental strategies and by using different markers for their isolation. However, there is still a lack of consensus on the phenotypic markers used in the isolation protocols of pure VSELs, requiring further direct functional comparison.

In addition, the main problem with the use of VSELs in regenerative medicine is their quiescence and limited number [17]. The ability of VSELs to expand in vitro is very limited, and requires a better understanding of their biology in order to stimulate their proliferative capacity without affecting their pluripotency. Unlike hematopoietic stem cells, where several methods have been tested to improve culture conditions, as a usage of cytokine combinations, feeder cell co-cultures or addition of recombinant proteins and small molecules [18–21], little is known regarding VSELs expansion process.

VSELs can, support vessel formation in vivo [22], and be specified to cardiomyocytes [23, 24] neurons [25] and hematopoietic stem cells [1, 26] both in vitro and in animal models. However, the precise molecular mechanism of how nascent VSELs control their pluripotency and differentiation potential remains to be determined. Kucia group, has previously demonstrated that highly purified murine bone marrow VSELs express a low level of mitotic genes and similar but not identical transcriptome to ESCs, which proliferate and differentiate normally [27]. When VSELs are induced to differentiate in co-cultures with a C2C12 supportive cell-line, a unique pattern in imprinted gene methylation is reverted that may explain in part VSELs quiescent status [28].

In the present study we highlight and characterize different populations of VSELs isolated from UCB on the basis of different markers. We then examine the transcriptome of VSELs expressing the pluripotent gene NANOG in order to ascertain the affected transcripts leading to their quiescence, to understand their biology and to establish ways to expand them in presence of a suitable medium. Interestingly, we have found that many proliferative genes have their expression affected in VSELs. In addition, we demonstrate that UM171, a pyrimidoindole derivative known to be able of inducing hematopoietic stem cells self-renewal [29], has a positive effect on diverse VSELs populations expansion and proliferation, i.e. CD133, CXCR4 and NANOG, increasing significantly their number without affecting their capacity to differentiate into organ-specific cells. These findings make VSELs a credible alternative of ESCs and induced pluripotent cells (IPS), as an easily available source of stem cells in regenerative medicine without ethical problems and undesirable side effects.

## Materials and Methods

### Isolation of VSELs and Flow Cytometry

All UCB samples were obtained from healthy persons, with informed consent and with the approval of local human subject research ethics boards (CED EFS, Besançon). Briefly, human UCB mononuclear cells were collected by centrifugation after reduction of red blood cells by Gelofusine (B Braun) treatment as described previously [30], followed by an additional red blood cell lysis with ammonium chloride lysis buffer (STEMCELL Technologies). Cells were then incubated with a cocktail of lineage specific antibodies directed against CD2, CD3, CD11b, CD11c, CD14, CD16, CD19, CD24, CD56, CD61, CD66b, and GlyA; from an EasySep™ progenitor cell enrichment kit with platelet depletion and human CD45 depletion kit (STEMCELL Technologies) for immuno-magnetic negative selection of Lin-CD45- cells using an EasySep™ magnet (STEMCELL Technologies). For NANOG VSELs isolation, SmartFlare™, Cyanine 5 fluorescent NANOG probe (Millipore) was added to the previously selected Lin-CD45- cells at the concentration of 10 μM. Next, the cells were incubated for 2 days in the indicated medium before VSELs staining and sorting.

Depending of the experiment, expanded VSELs or not were then stained with a mixture of lineages (Lin) associating monoclonal antibodies (MoAbs) conjugated with fluorescein isothiocyanate (FITC). At the same time, V500 conjugated-CD45 (Beckman Coulter), CD34 PE clone 8G12 and a combination of allophycocyanin (APC) conjugated MoAbs, CD133 clone AC133 (Miltenyi Biotec, Paris, France), or CD184 (CXCR4) clone 12G5 (BD Biosciences), were added for 30 min on ice. For NANOG+ VSELs isolation, the cells were pre-cultured for 2 days with SmartFlare™, Cyanine 5 fluorescent NANOG probe (Millipore). Cells were then washed and discriminated by flow cytometry on the basis of cell size, granularity, and presence of CD34, absence of Lin and CD45 markers. Then, depending of the VSELs population studied, presence of NANOG, CD133 and/or CXCR4 markers were gated. Cells viability was monitored by the absence of dye 7-AAD (BD Biosciences) uptake which was added 10 min before acquisition. All flow cytometry sorting or analysis was performed using a BD ARIA III instrument (BD Biosciences). Data acquisition and analysis was conducted using BD FACSDiva software (BD Biosciences).

### RNA-Seq Data Processing

EasySep™ Lin-CD34 + CD45- purified cells were cultured in conditioned media in presence of SmartFlare™, fluorescent probe (Millipore) for 2 days. Then, 300 sorted VSELs expressing NANOG and control cells negative for NANOG expression were used for cDNA synthesis with the help of Smarter ultra-low input RNA kit for sequencing (Clontech). Sample quality was assessed using Bioanalyzer RNA Nano chips (Agilent). Paired-end, barcoded RNA-Seq sequencing libraries were then generated using the Nextera XT DNA library preparation Kit (Illumina) following the manufacturer’s protocols. The quality of library generation was then assessed using a Bioanalyzer platform (Agilent), and Illumina MiSeq-QC run was performed or quantified by qPCR. Sequencing was performed using an Illumina HiSeq2500 using TruSeq SBS v3 chemistry at iGE3 Genomics Platform (University of Geneva). The normalization and differential expression analysis was performed with the R/Bioconductor package edgeR v.3.10.5, for the genes annotated in the reference genome. The raw count data are filtered. We filter out very lowly expressed genes, keeping genes that are expressed at a reasonable level. We keep genes that achieve 10 counts in at least 2 samples. The filtered data are normalized by the library size and differentially expressed genes are estimated using the GLM approach (Generalized Linear Model).

The fold change (FC) of base 2 logarithm of the trimmed mean of M-values normalization method (TMM normalized data) log_2_ FC was used to rank the data from top upregulated to top downregulated genes and FDR (0.05) was used to define significantly differentially expressed genes. RNA-Seq results are accessible in supplementary data 3.

### Cell Cycle and Apoptosis Assays

Cell cycle progression was monitored with vybrant dyeCycle violet kit (Invitrogen). Briefly, VSELs stained with the appropriate antibodies at the indicated time of culture and in the indicated media, were incubated for 1 h at 37 °C at a final concentration of 10 μM of vybrant dyeCycle violet to labelled DNA. Then 7AAD was added 10 min before FACS acquisition to exclude died cells. For apoptosis analysis, Annexin V PE (Invitrogen) and 7-AAD (BD Bioscience) staining of day 14 suspension cultures was performed according to the manufacturer’s protocol.

### Real Time RT–PCR Analysis

For all real time RT–PCR determinations, total cellular RNA was isolated with RNAeasy kit according to the manufacturer’s instructions (QIAGEN) and cDNA was synthesized using the iScript cDNA Synthesis Kit (BIO-RAD, France). Real time RT-PCR was done in triplicate with SYBR® Green PCR Supermix (BIO-RAD). The mRNA content of samples compared was normalized based on the amplification of GAPDH and/or *β*2-microglobulin. The oligonucleotides used for real time RT-PCR genes amplification from human UCB are listed in supplementary Table 1. The relative quantification value of target was calculated according to the formula: 2^-^ΔΔ^Ct^, where ΔCt = Ct of target genes - Ct of endogenous control gene, and ΔΔCt = ΔCt of calibrator - ΔCt of samples. For this method to be valid, equivalent efficiencies between target gene and endogenous control gene PCRs were presumed.

### VSELs Expansion

Gelofusine and EasySep™ treated UCB cells were cultured for 10 to 12 days in StemSpan™ ACF medium (STEMCELL Technologies) supplemented with growth factors, Stem Cell Factor (SCF; 100 ng/ml, R&D Systems), Flt3 ligand (FLT3-L; 100 ng/ml, R&D Systems) and Thrombopoietin (TPO; 20 ng/ml, PEPROTECH). When indicated, UM171 (35 *μ*M, STEMCELLS Technologies) and/or SR1 (500 *μ*M, STEMCELLS Technologies) were added. Viable Lin-CD34+CD45-CD133+, Lin-CD34+CD45-CXCR4+ or Lin-CD34+CD45− NANOG+ cells were then sorted and quantified.

### VSELs Differentiation

For mesoderm and endoderm differentiation, we first used according to the manufacturer’s instructions, STEMdiff™ Trilineage differentiation Kit (STEMCELL Technologies) described as rapid test which provides a simple culture assay to functionally validate the ability of human embryonic stem cells and induced pluripotent stem cell lines to differentiate to germ layers and is intended to be an endpoint rapid assay allowing determination of cells potential differentiation within one week. We then also used with some modifications a specific culture medium (MV06™) allowing cell differentiation to endothelial and cardiac destinies and previously established in our laboratory [31]. Briefly, the sorted VSELs were incubated on day 0 on fibronectin/gelatin-coated 96-well plate in this medium. On day 2 and day 3, the cells were treated with 2.5 mM valproic acid (VPA), and 0.5 μM of 5-azacytidine (AZA) respectively, half of the medium was then renewed every 2 days with medium containing 10 nM of ascorbic acid and 1 ng/ml of TGF-β until day 14.

### Clonogenic Progenitor Assays and Hematopoietic Differentiation

Human clonogenic progenitor cell assays were done in semi-solid methylcellulose medium Methocult H4434; (STEMCELL Technologies) with flow-sorted 12 days expanded VSELs (50 cells per ml). Colony counts were carried out after 14 days of incubation at 37 °C and 5% CO2. In parallel, 50 to 100 cells/ml were cultured for 15 days in DMEM containing 10% fetal calf serum, glutamine, penicillin/streptomycin and 50 ng/ml of SCF, IL3, 1 U/ml of erythropoietin.

### Statistical Analysis

All data are presented as average ± SD of at least 3 independent experiments, unless specifically mentioned. Student’s *t* test was applied for statistical analysis, as appropriate. *P* values of <0.05 were considered significant.

## Results

### Characterization of Markers Expression in VSELs Subpopulation

Typically, VSELs are purified on the basis of the CD34 extracellular receptor expression and the exclusion of hematopoietic and mature cells expressing CD45 receptor and/or positive for the expression of lineage markers. Other additional criteria as the CD133, or CXCR4 receptors expression were used to identify and isolated these pluripotent stem cells [15, 32]. This led to the description of different types of VSELs, the identity of which remains to be determined. To resolve the ambiguity about the nature of these different populations, described in the literature, we have performed cells surface receptors multi-labeling and used NANOG mRNA expression as an additional new criterion in order to discern the overlapping VSELs and then isolate and characterize them individually. We therefore, labelled and isolated the following three categories of VSELs which diverge between them by a single marker, CXCR4, NANOG or CD133 expression:

$$ {\displaystyle \begin{array}{l}\mathrm{Lin}-\mathrm{CD}34+\mathrm{CD}45-\mathrm{CD}133+\mathrm{NANOG}+\\ {}\mathrm{Lin}-\mathrm{CD}34+\mathrm{CD}45-\mathrm{CD}133+\mathrm{CXCR}4+\\ {}\mathrm{Lin}-\mathrm{CD}34+\mathrm{CD}45-\mathrm{NANOG}+\mathrm{CXCR}4+\end{array}} $$

**Fig. 1:**
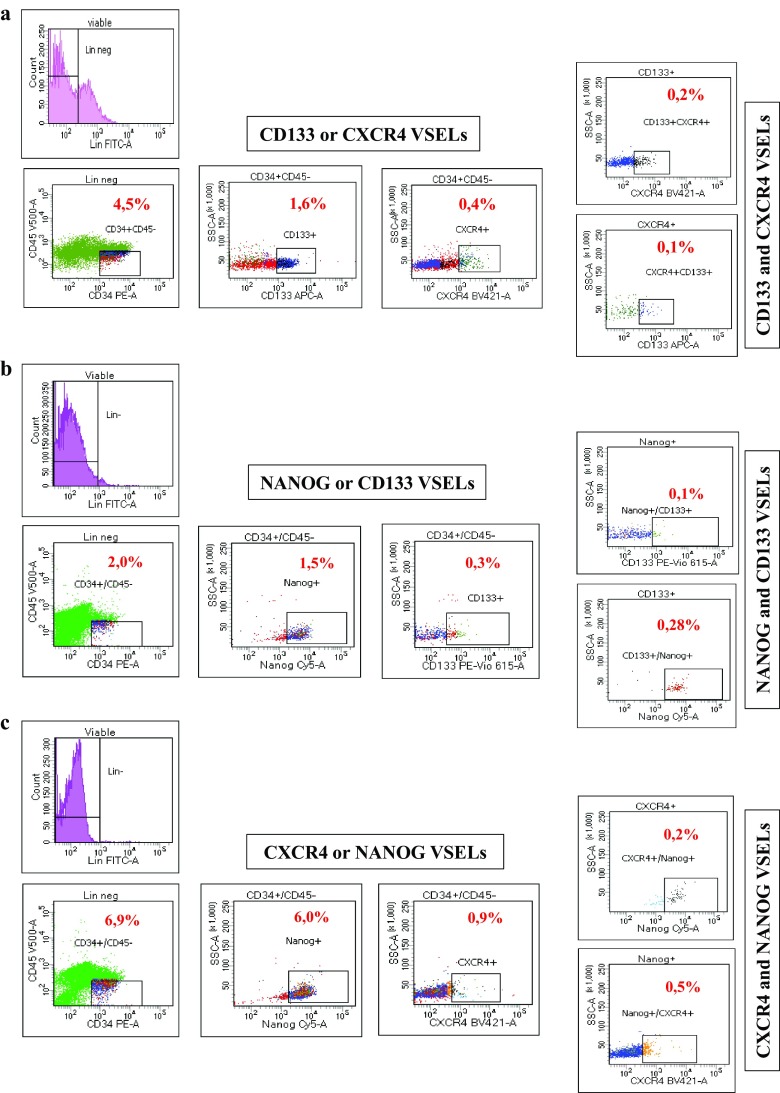
Cord blood VSELs surface markers and NANOG mRNA multi-labeling. Three categories of stem cells present in UCB are labeled with the indicated antibodies and analyzed by flow cytometry. These three populations diverge between them by a single marker, and are thought to represent VSELs **a** Lin-CD34 + CD45-CD133 + CXCR4+ **b** Lin-CD34 + CD45-CD133 + NANOG+ **C** Lin-CD34 + CD45-NANOG+CXCR4+. The percentages of VSELs among nucleated cells are indicated in red, and show that different subpopulations of VSELs are present in cord blood in terms of markers expression (representative experiment)

Flow cytometry analysis showed that Lin-CD34 + CD45- cells expressing CD133 represent 1.6% of total cells while those expressing CXCR4 represent only 0.4% (Fig. 1a). However, among these CD133 VSELs only a part of them express also CXCR4 marker (0.2% of total cells). Similarly, CXCR4 VSELs expressing CD133 receptors represent only 0.1% of total cells. These results clearly demonstrate that there are several subpopulations of VSELs that may contain cells lacking at least the expression of one marker or that the extents of described VSELs in the literature are overestimated by additional isolation of non-related cells. This finding is confirmed in our second analysis using NANOG instead of CXCR4, which also shows the presence of 1.5% and 0.3% of VSELs Lin-CD34 + CD45- expressing NANOG or CD133 respectively, whereas double positive cells for these two markers are less than 0.3% (Fig. 1b). These discrepancies were observed also when we studied populations expressing NANOG or CXCR4 alone or both markers (Fig. 1c). In the light of these results, VSELs are generally isolated based on Lin-CD34 + CD45- cells expressing CD133 or CXCR4 receptor alone, rarely on their combination, suggesting that VSELs populations are overestimated during isolation. We considered afterwards that those expressing the pluripotency specific gene NANOG might be close to embryonic stem cells and more suitable for our further molecular investigations.

### The Whole Genome Transcripts of VSELs Study

**Fig. 2:**
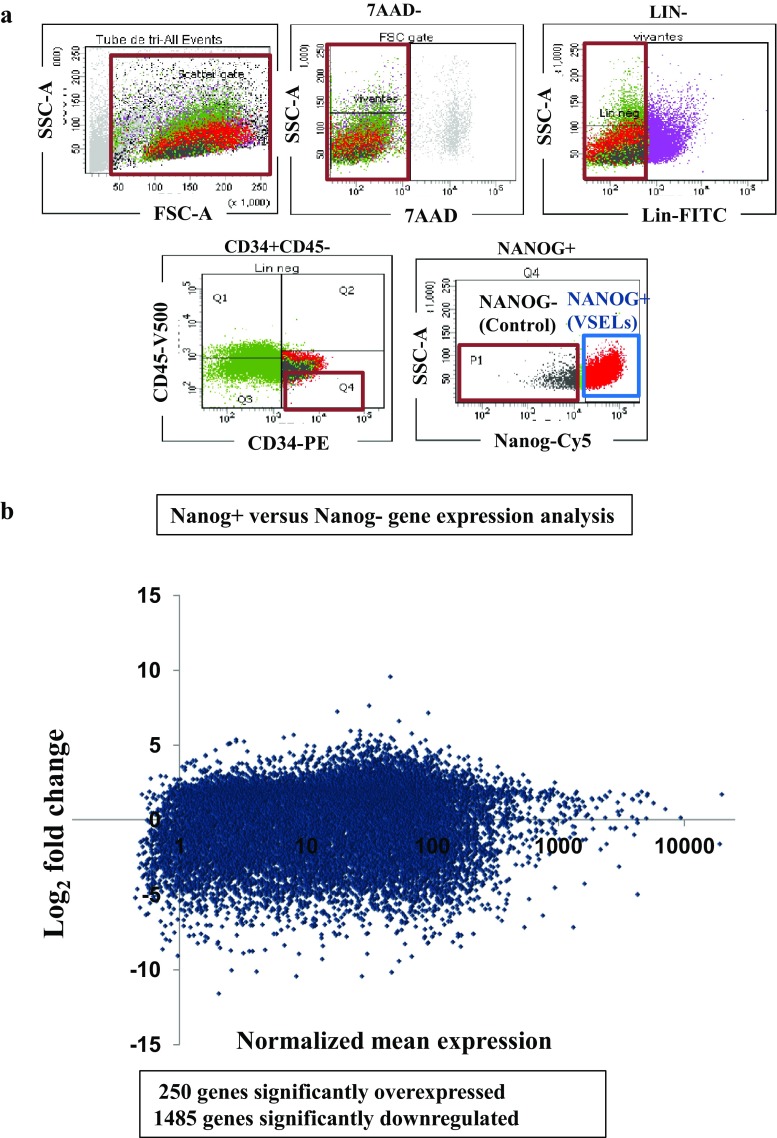
Sorting profile of VSELs and control cells used in RNA-Seq and their High-throughput expressions. **a** FACS profile of viable Lin-CD34 + CD45- expressing NANOG mRNA are referred as VSELs and NANOG- cells representing the control cells (The red boundary regions represent the populations containing VSELs or the control cells). **b** Differential expression profiles of 19,114 genes determined by RNA-Seq. The NANOG+ versus NANOG- gene expression Scatter plot, representing log_2_ of their normalized read counts ratio calculated from the means of two cDNA libraries. About, 250 genes are significantly upregulated and 1485 are significantly downregulated

Quiescence and scarcity of VSELs make them difficult to use as they are in cell therapies, thus it is necessary to purify them and induce their proliferation. We first improved VSELs isolation by looking for the purest population (positive for NANOG expression) in order to dissect the molecular processes governing their growth. We then, have sought a possible discrepancy in genes expression with standard embryonic stem cells, which proliferate and differentiate normally. Therefore, by flow cytometry sorting, we isolated VSELs on the basis of embryonic and pluripotent cells specific NANOG gene mRNA expression, labeled by the SmartFlare™ fluorescent probes, and a control population not expressing this gene (Fig. 2a). The transcriptome of these two populations were then compared.

To characterize the earliest transcriptional difference, we performed RNA sequencing to quantify the whole transcriptome of these two populations at day 2 after purification from UCB and carry out a statistical study of differential gene transcription between NANOG VSELs and control cells. This allowed us to identify genes repressed or overexpressed in VSELs with respect to a control cell population. We were able to determine the expression levels of 19,114 genes (approximately 81% of the transcriptome) (Fig. 2b). Among these genes, 1736 are significantly differentially expressed by at least two logarithmic factors. The number of genes, the expression of which is increased was 250, while 1485 repressed genes were found. This finding demonstrates that there are many more repressed genes in VSELs than in control cells which might explain their quiescence.

**Fig. 3:**
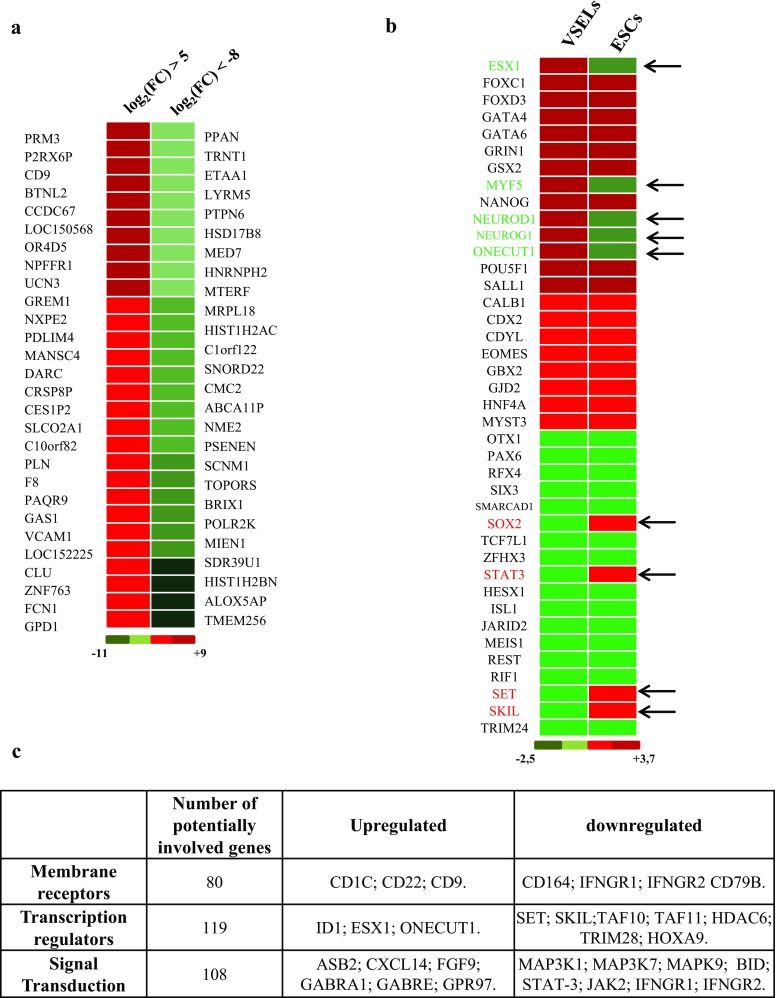
Differential expression of VSELs mRNA. **a** Heat map showing in red the main induced genes in VSELs by comparison to control cells (log_2_ fold change higher than five), and the most downregulated gene in green (log_2_ fold change higher than eight). **b** Heat map showing a comparison between gene expression variations in VSELs relative to control cells (present study) and those observed regularly in embryonic stem cells during maintenance, arrows annotated the genes with a significant discrepancy (green is a decrease and red is increase relative to control). The color scale is shown at the bottom. **c** Classification by their function or their localization of the differentially expressed genes observed by RNA-Seq

The identification of the most strongly increased or severely diminished genes does not show genes known as implicated in the control of pluripotent cells quiescence and differentiation. However, membrane receptors such as CD9 and CD22 appear to be greatly increased as well as Protamine 3 (PRM3), whereas TMEM256, a membrane protein mRNA and histones HIST1H2AC, HIST1H2BN mRNA remain among the most decreased transcripts (Fig. 3a), assigning them a possible role in VSELs development and/or quiescence. We then looked at the expression levels variation described in the literatures of genes known as regulators of ESC pluripotency, and found discordance in these variation levels in VSELs when compared to those of ESC. As an example, MYF5, NEUROD1, NEUROG1 and ONECUT1 were upregulated in NANOG VSELs under steady state condition (Fig. 3b) while several studies have shown that their expression is low in ESC [33, 34]. In contrast, some of the transcription factors critical in the undifferentiated phenotype maintenance of ESC and promoting self-renewing, like SOX2, SKIL, SET and STAT-3 [35–37], have their transcripts down regulated in VSELs.

Analysis of gene panels variation according to their functions show that some sets of genes expression are affected in NANOG VSELs. These gene clusters include cell receptors, kinases, genes that control the cell cycle; others are involved in the transduction of cell signal from extracellular environment, or DNA repair. We have represented in the table (Fig. 3c) the genes, differentially expressed in significant levels, and classified them according to their functions and locations. We have thus seen that receptors (80 genes), around 119 transcription regulators and 108 cell signaling transducers have their expression up or downregulated in VSELs. Interestingly, genes known as proliferation and survival enhancers such as HOX9, MAP3K1, MAP3K7, and MAPK9 are much less expressed in VSELs compared to the control cells (Fig. 3c). The expression of some of these genes was validated by real time RT-PCR in order to determine a set of genes, involved in the quiescence of VSELs. We can then consider their activation through growth factors to stimulate self-renewal.

**Fig. 4:**
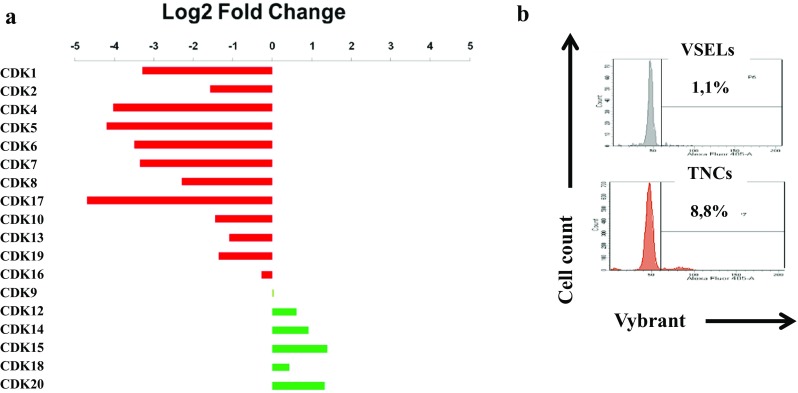
Differential expression of Cyclin Dependent Kinases mRNA and cell cycle stage in non-expanded VSELs. **a** CDKs mRNA expression determined by RNAseq experiment in VSELs represented as Log_2_ fold change by contrast to control cells. **b** Cell cycle determination by vybrant labeling of VSELs and total nucleated cells (TNCs), after 24 h culture in minimal media (representative experiment)

Another group of genes, very important in the control of proliferation, includes several cyclin dependent kinases (CDKs). These kinases are known to associate with cyclins and allow their phosphorylation. The cell cycle is controlled by at least 6 different cyclins / CDK complexes which mediate at specific moments the cell cycle. The analysis of CDKs expression in VSELs revealed that the most important of them (CDK1 to CDK8) have their expression highly affected (Fig. 4a). This suggests that their low expression in VSELs would be responsible for the cell persistence in G0/G1 phase as observed by the cell cycle analysis of Vybrant labeled CD113+ VSELs (Fig. 4b) and would explain the observed quiescence. This analysis shows that significantly lower percentages of VSELs cells are in the G2M phase (1.1%) under steady state condition in comparison to total nucleated cells (8.8%). Strategies to activate their proliferation can be settled in order to stimulate the cell cycle entry of VSELs.

### VSELs Expansion

Recently, through high-throughput culture experiments, a pyrimidoindole derivative, UM171, able of amplifying CD34+ hematopoietic stem cells, has been identified among 5280 small molecules tested [22]. As VSELs express also this receptor, we decided to investigate its effect on these pluripotent cells; in parallel, we compared its ranges to an aryl hydrocarbon receptor antagonist, the StemRegenin1 (SR1) which is also known as promoting the expansion of UCB stem cells [17].

**Fig. 5:**
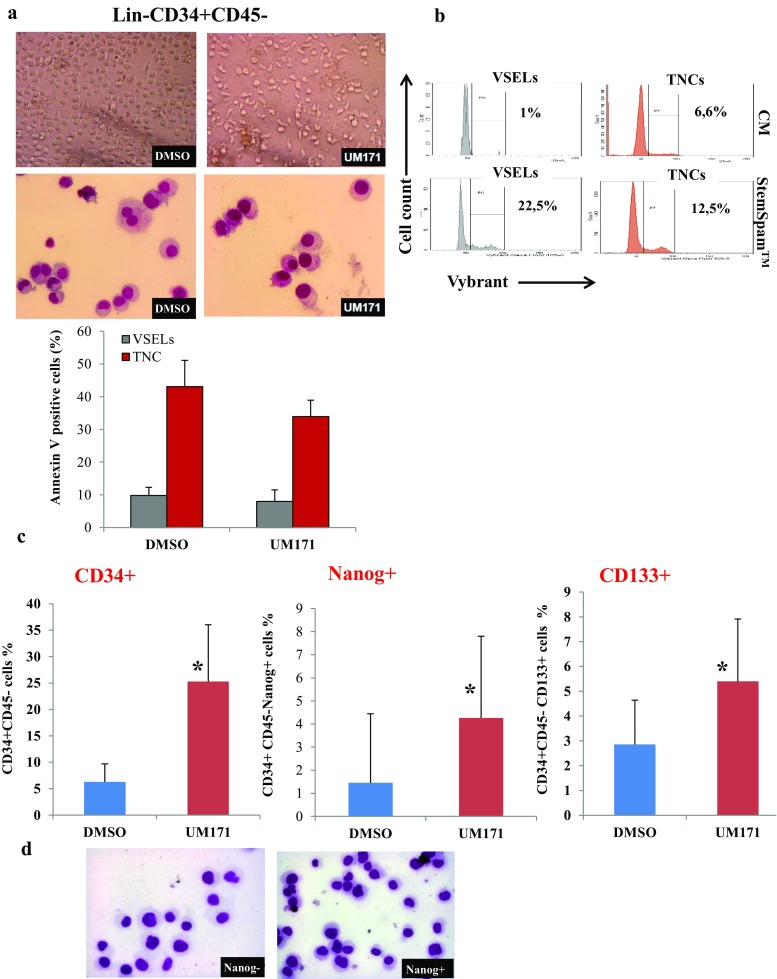
Expansion of VSELs and their characterization. **a** The EasySep™ purified Lin-CD34 + CD45- cord blood cells morphology as observed by optical microscopy (upper panels) or their May-Grünwald Giemsa cytospin preparations (lower panels) at day 9 cultures in media supplemented with DMSO as control or UM171. Annexin V staining of VSELs and total nucleated cells (TNC) after 14 days culture in control media or supplemented with UM171 are shown on the bottom. **b** The 7AAD + Lin-CD34 + CD45-CD133+ VSELs cell cycle determination by vybrant labeling after 24 h expansion in StemSpan™ containing UM171, in comparison to VSELs cultured in minimal media (conditioned media CM). In the right panels are represented the cell cycles determined from the total nucleated cells (TNCs) in the same conditions of culture than VSELs (representative experiment). **C** The percentages of CD34+ stem cells and phenotypically defined VSELs subsets NANOG+ and CD133+ calculated after 12 days expansion of Lin-CD34 + CD45- cells. Bars represent average ± SD, *n* = 4 (*P < 0.05). **d** May-Grünwald Giemsa cytospin preparations of sorted NANOG+ and NANOG- VSELs at day 12 of expansion

**Fig. 6:**
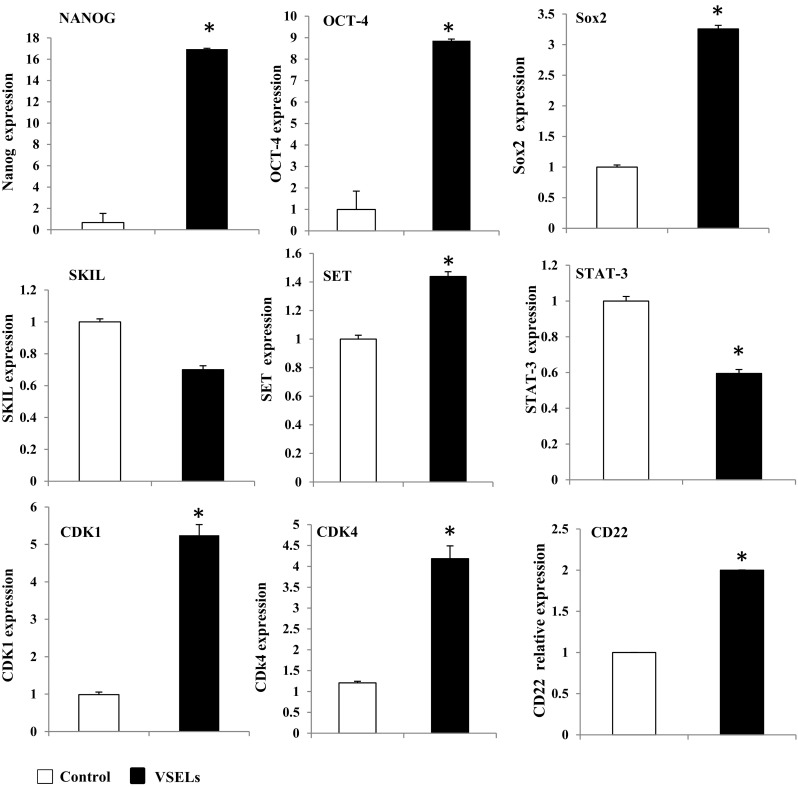
Gene expression analysis of expanded VSELs. Relative expression analysis of the indicated genes in CD133+ VSELs or control cells after expansion was determined by real time RT-PCR. Data are presented as means ± SD for three individuals. *P < 0.05

To test this hypothesis, we sorted Lin-CD34 + CD45- cells and settled in agreement with RNA-Seq mRNA expression results, strategy to activate the expression of the affected genes in VSELs, by selecting a culture medium devoid of any animal components and supporting stem cells self-renewal. This had led us as described in materials and methods to grow VSELs in StemSpan™-ACF medium (STEMCELL Technologies) in the presence of growth factors and UM171 or DMSO as control. Observation by microscopy shows that the cells are able to survive and proliferate under both conditions (Fig. 5a). Nevertheless, in comparison to the control cells, UM171 treated VSELs remain mononuclear by maintaining undifferentiated morphological features even after 12 days of culture. Apoptotic cells examination by annexin V labelling, show no significant differences between UM171 treated VSELs and the control cells (lower panel). The VSELs cell cycle analysis show that the culture in this new medium increases significantly the percentage of cells in G2M stage (22.5% in StemSpan™ containing UM171vs 1% in conditioned media) (Fig. 5b). In contrast, the effect is less apparent on treated total nucleated cells (TNC). Analysis of the VSELs extent after twelve days of incubation in these media by cytometry confirms the ability of UM171 to amplify the CD34 + CD45- cells. Interestingly, quantification of VSELs expressing the labeled NANOG mRNAs showed an increase in the percentage of these cells when UM171 is present compared to control, demonstrating that this molecule has a positive effect on VSELs self-renewal. Conversely, SR1, despite its positive effect (but to a lesser degree on the expansion of CD34+), does not increase the NANOG+ population (data not shown). However, a percentage of VSELs, relative to total nucleated UCB cells, shows that UM171 allow their amplification by 13-fold (0.2% DMSO vs. 2.6% in the presence of UM171). In addition, FACS analysis of VSELs CD34 + CD45-CD133+ shows that their number also is increased in presence of media containing UM171 in comparison to control cells highlighting its positive effect on this subpopulation (Fig. 5c). These data were confirmed by the absolute numbers determination of Lin- CD34+ cells and VSELs by using the BD Trucount™ after 12 day of expansion (Supplementary Fig. 1). Nevertheless, once NANOG+ VSELs were expanded, their size become comparative to control cells in agreement with the fact that the transition state between proliferation and quiescence is frequently associated with changes in gene expression, extent of chromatin compaction, and histone modifications (Fig. 5d). Finally, gene expression analysis in 12 days expanded VSELs indicates that some key regulator genes downregulated during steady state condition, observed in the RNA-Seq study, becomes induced in comparison to control cells once VSELs self-renewal is prompted. Indeed, in contrast to STAT-3 and SKIL, VSELs expansion is associated with the induction of SOX-2 and SET mRNA expression as shown by real time RT-PCR in (Fig. 6). Interestingly, VSELs keeps the induced expression of the genes implicated on their pluripotency, such as NANOG and OCT4. In addition, CDK1 and CDK4 expression is restored and become induced by 4 to 5 folds when VSELs are under expansion (Fig. 6).

**Fig. 7:**
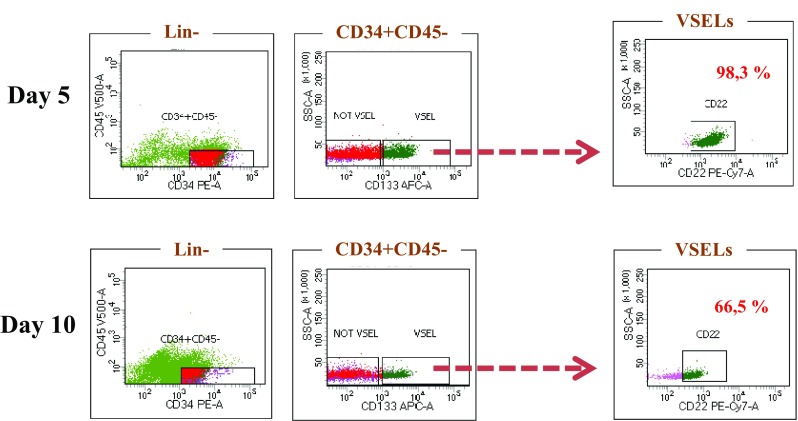
Differential expression of CD22. FACS quantification of CD22 expression on day 5 and day 10 showing a strong expression of CD22 in the VSELs (representative experiment)

The discovery of a receptor expressed exclusively on VSELs surface, would still be a great finding which could simplify and reduce the cost of pure VSELs isolation without NANOG mRNA labeling. Thus expression of CD22 by real time RT-PCR (Fig. 6) confirms the high expression of this receptor on VSELs, and Flow cytometry analysis shows that around 98% of them are CD22 positive on day 5 of expansion (Fig. 7). However, 66.5% still remain positive on day 10 of expansion. Our identification of this gene encoding additional VSELs-enriched surface receptors will facilitate the isolation of these cells using fluorescent antibodies.

### Expanded VSELs Differentiation

**Fig. 8:**
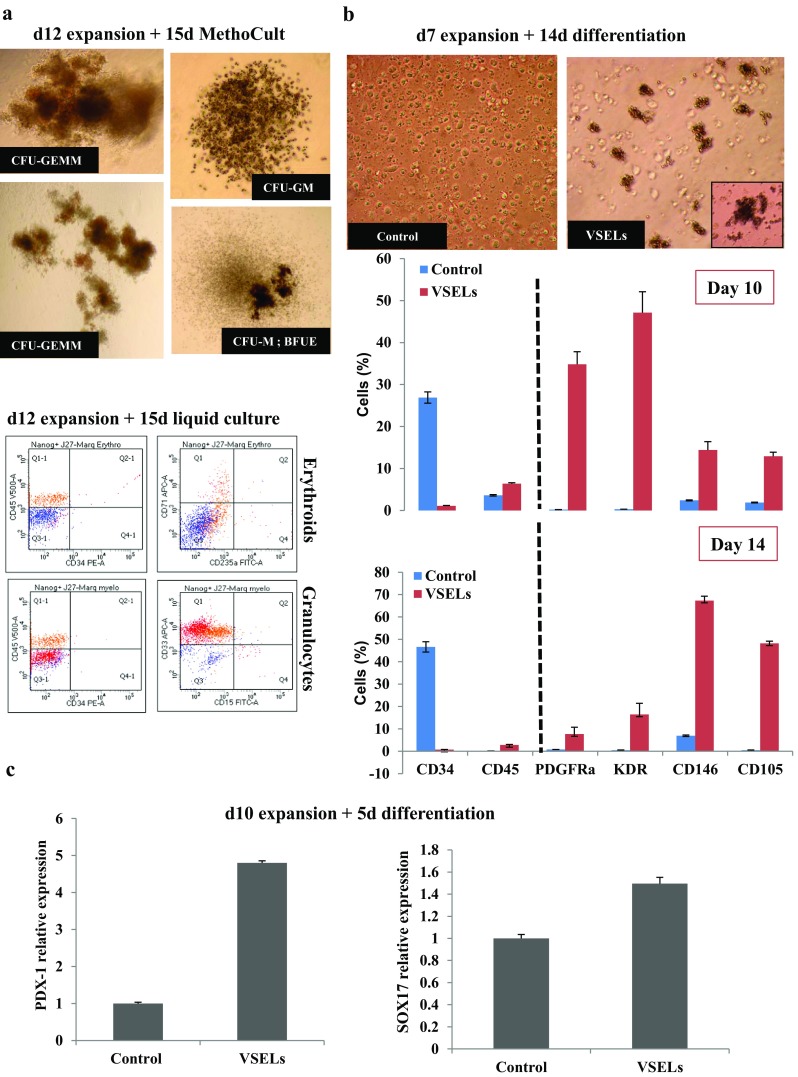
Expanded VSELs differentiation. **a** Hematopoietic colonies formation during 15 days in MethoCult from the sorted Lin-CD34 + CD45-NANOG+ VSELs which were previously expanded 12 days in culture medium containing UM171 (Upper panels). FACS analysis of hematopoietic cells formation from 15 days culture of Lin-CD34 + CD45-NANOG+ VSELs after 12 days expansion in culture medium containing UM171 (Lower panels). **b** The VSELs and control cells morphologies after 10 days of expansion and 14 days of mesoderm differentiation (upper panel). Phenotypic analysis by flow cytometry of PDGFR*α*, KDR, CD146 and CD105 mesoderm markers in VSELs and controls cells induced to differentiate in MV06™ media for 10 and 14 days. Bars represent average ± SD, *n* = 3 (*P < 0.05). **c** SOX17 and PDX-1 endodermic markers mRNA expression by real time RT-PCR of Lin-CD34 + CD45-CD133+ VSELs and control (CD133-) cells that were previously expanded for 10 days and induced to differentiate 5 days in endodermic medium.

We then tested the ability of VSELs, thus amplified in presence of UM171, to differentiate towards the different germ cells layers. For hematopoietic destiny, VSELs expanded for 12 days, were placed in presence of culture medium supporting this cell type differentiation. We observed that 15 days culture in methylcellulose or in liquid medium, containing SCF, IL3 and erythropoietin, (growth factors promoting hematopoietic differentiation), allow respectively the formation of most colonies CFU-GEMM, CFU-M and BFU-E (Fig. 8a) upper panel, and both myeloid (CD45+, CD15+, CD33+) and erythroid cells (CD71+, CD235a + = glycophorin A) (Fig. 8a) lower panel.

The mesoderm is one of the three embryonic layers, responsible for the formation of hematopoietic as well as, bone, endothelial and cardiac cells. As we have been able to differentiate VSELs to form blood cells in the presence of appropriate medium and growth factors, we looked for culture media that favor the endothelial cells formation. We therefore first cultured the 10 days expanded and sorted CD133+ VSELs on STEMdiff™ Trilineage mesoderm culture medium known to be able to promote the ESC and IPS cells differentiation towards mesodermal lines in order to test its effects on VSELs differentiation. Our results indicated unfortunately that only few cells express endothelial markers and are committed to differentiate (Supplementary Fig. 2). This medium is not optimized for the generation of cells for downstream differentiation but can inform easily and in the short time on the capacity of VSELs to differentiate. We then used a specific culture medium (MV06™) which we have previously shown able to allow mobilized CD34+ cells to differentiate into both the endothelial and cardiac muscle cell pathways [31]. After ten and fourteen days of culture in sequential presence of VPA, AZA and TGF-β as described in materials and methods, the differentiated VSELs have formed embryoïd bodies like structures that were collected and in which, the expression of endothelial markers PDGFRα, CD309(KDR), CD146 and CD105, were measured. As shown in (Fig. 8b), expression of these receptors is significantly increased in the cultures containing VSELs relative to the control cells. Conversely, the number of CD34+ stem cells decreased severely reaching only 0.7% and hematopoietic CD45 positive cells still insignificant. The expression of PDGFRα and KDR appeared at significant levels on day 10; four days earlier than CD146 and CD105 (Fig. 8b), in agreement with previous studies in which these two markers were used in combination to permit enrichment of cardiac progenitor cell populations from pre-cardiac mesoderm induced ESC differentiation [38, 39]. Indeed, once sorted and exposed to specific conditions, the PDGFRα, KDR cells will give rise to more mature pericyte progenitors CD105+/CD146+/CD73+/CD133+. These results are very promising and show that VSELs still have pluripotent cell features capable of forming cells with endothelial characteristics even after a pronounced expansion.

We then stimulate the expanded VSELs for 5 days to differentiate toward endodermic fate in STEMdiff™ Trilineage endoderm medium according to the manufacturer’s instructions. As shown in (Fig. 8c), this pluripotency was confirmed by the appearance of a higher PDX-1 and SOX17 mRNA expression from VSELs cultures in comparison to controls.

## Discussion

As much as accessibility, appearance of teratomas and ethical problems related to usage of human embryos or IPS as a therapeutic tool in regenerative medicine still difficult to solve [40, 41], VSELs remain an excellent alternative of stem cells source on condition that their isolation, proliferation and differentiation are mainly controlled. This can only be achieved through a better understanding of their biology and the molecular mechanisms governing their quiescence. For this reason, we first attempt to look for pure populations and were able to isolate different VSELs on the basis of various markers described in the previous studies of VSELs [10, 15, 32], alone or in combination, such as the CD133 receptor, the CXCR4 receptor, or for the first time, NANOG gene expression. We have observed that several populations of VSELs may exist; we then characterized their potential of expansion and differentiation. Our results, show that VSELs, Lin-CD34 + CD45- expressing CD133+ or NANOG+ have the same expansion and differentiation capacities towards the mesodermal and endodermal pathways. We continued these experiments by determining differentiation capabilities of VSELs which express the CXCR4 marker, our preliminary results show that they have less ability to proliferate and differentiate in our media. VSELs that were not sorted exactly on the same markers show also different levels of differentiation propensities in vitro or in vivo as well as organ repair capacity, supporting these data [17, 26, 42]. We concluded that VSELs expressing the Nanog gene would be the most representative of pluripotent cells and then extended to them the molecular studies.

VSELs transcriptome investigation by new generation sequencing method allowed us the mapping of their whole genome, and the identification of some genes governing their quiescence. As previously described in murine bone marrow VSELs [43], we found that some genes implicated in proliferation control were down-regulated in VSELs isolated from UCB, as cyclin and signal transduction genes. We corroborate by real-time PCR that most cyclins expression is affected in VSELs and identified new genes downregulated as shown in (Fig. 3c). This type of regulation is known to poise the cells on proliferation arrest [44] and may explain the difficulty of making VSELs proliferate in the usual culture media, issue that we have addressed later by a selection of suitable media for induction of their self-renewal and proliferation and then media for their differentiation.

We have also been able to demonstrate that it is possible to multiply VSELs and have them in large numbers (10 to 15 fold expansion) by using small molecule, suitable media and growth factors. Their expansion is an essential step in order to extend the study further towards the understanding of their functioning and envision their large-scale use in stem cells therapy. However, in cultures of human ESC, grown under conditions that permit stem cell renewal, a continuous gradient of expression of genes associated with pluripotency was observed [45]. Interestingly, we observed that the expression of NANOG and OCT-4, still sustained after expansion and that some genes encoding for a key regulators of stem cells maintenance and pluripotency are enriched in VSELs after expansion. Indeed, SOX2 and SET which were downregulated under steady state condition in comparison to controls, become upregulated in contrast to SKIL and STAT-3, assigning them a possible role in VSELs expansion. Studies using High-throughput sequencing (ChIP-Seq) to map DNA binding locations of the core and accessory pluripotency factors, on a genome-wide scale, revealed that these two genes have a higher propensity for binding many regulatory regions of essential developmental genes [46, 47]. Previous study on hematopoietic stem cells have shown that UM171 suppress transcripts associated with erythroid and megakaryocytic differentiation and that the most highly up-regulated genes in UM171-treated cells encode for surface molecules such as the transmembrane protein of unknown function, TMEM183A and endothelial protein C receptor (EPCR/CD201/PROCR), a known marker of mouse LT-HSCs and UM171-expanded human cord blood HSCs. [29, 48]. In line with these results, these two genes expression seem to not be significantly affected on VSELs. However, global gene expression signatures of VSELs exposed or not to UM171 should be studied in order to have more insight on UM171 mode of action.

On the other hand, we confirm both by PCR and flow cytometry that the CD22 receptor was strongly expressed in VSELs. Identifying this new marker may allow its employment to isolate VSELs with high purity, question that we addressed and is under investigation. However, CD22 receptor function in these pluripotent stem cells remains to be determined.

We obtain for the first time evidence that UCB VSELs are able to form different tissues even after 10 to 12 days of expansion, keeping their ability to form hematopoietic colony forming unit, erythroid and myeloid cells. We have acquired STEMdiff™ Trilineage culture media known for their ability to stimulate ESC differentiation towards the mesoderm, ectoderm and endoderm. The first tests showed that VSELs, unlike the controls, are able to differentiate and express endothelial markers after 5 days of culture in the mesodermic medium. However, the level of differentiated cells remains quite limited. Nevertheless, using such medium keep staying interesting by the fact that it can serve as rapid potency test, able to determine the capacities of the sorted VSELs to give rise to the 3 germ layers. Importantly, we established a new culture condition improving VSELs differentiation to mesoderm, in MV06™ medium [31]. The number of positive cells for endothelial markers is remarkably improved in comparison with other studies even if some cells still refractory to differentiation. We postulated that this could be due to the incidence of subpopulations of VSELs phenotypically different as we demonstrated in the present study and shown in (Fig. 1). On the other hand, the isolated VSELs could possibly be at different stage of pluripotency and might have a varied potential to give rise to one specific lineage. Low extents of differentiation were observed recently even with mouse bone marrow VSELs [42], cells that never exceed an overall differentiation of 5 to 13% into one of the three germ layers. In addition, in order to demonstrate that VSELs have perspectives as source of cells in regenerative medicine, we looked only for culture condition restricted to animal free component which likewise may restrict the number of differentiated cells in comparison to culture on feeder cells. Indeed, before VSELs are transferred to the differentiation medium, they are initially cultured on feeder cells for a few days [7] and not directly in the expansion medium as it are a case in the current study. It is now necessary to further improve the purity of VSELs using new markers and continue these differentiation tests with other subpopulations of VSELs.

The study of normal development of stem cells revealed that, in addition to classical genetics, regulation of gene expression is also affected by “epigenetic” modifications, such as chromatin remodeling and histone variants, methylation of DNA, regulation by proteins of the Polycomb group reviewed by Orkin et al.; [49], and the epigenetic function of non-coding RNAs [50, 51]. To further understand the quiescence of VSELs and the events likely to trigger their proliferation and differentiation, it is necessary to explore how these genetic and epigenetic marks change VSELs destiny by regulating the expression of genes, questions that are addressed in our laboratory.

We also estimated that it is necessary to test the ability of these expanded stems cells to reconstruct damaged tissues in vivo in mouse or rate models to confirm their pluripotency. We can then consider by using growth-factor and/or molecule-based treatments in a reasonable and pharmacological way stimulating these cells and prompting their proliferation and/or differentiation to form different tissues capable of repairing injured organs.

## Conclusions

The treatments of a number of incurable diseases, such as leukemia, become possible through stem cells transplantation with exciting results. However, use of stem cells for solid tissue transplantation has not been met with similar success [40]. Issues with safety and production efficiency have retarded the progress and clinical applications of stem cell therapy by ESC and IPS by the fear of gene mutation and tumor development [41]. In the present study, we have attempted to clarify VSELs biology and develop their potential application as “natural” source of stem cells, by finding a way simplifying their expansion and differentiation, and by consequence stem cell therapy.

## Electronic supplementary material

ESM 1 (PPTX 63 kb)

ESM 2 (XLSX 3130 kb)
