# Supplementary material for: VSELs Maintain their Pluripotency and Competence to Differentiate after Enhanced Ex Vivo Expansion
Source: Stem Cell Rev. 2018 May 8;14(4):510–24. doi: 10.1007/s12015-018-9821-1 (PMC6013546; doi:10.1007/s12015-018-9821-1)
Supplement: Supplementary file 1 — (PPTX 63 kb) [file 12015_2018_9821_MOESM1_ESM.pptx]

## Slide 1
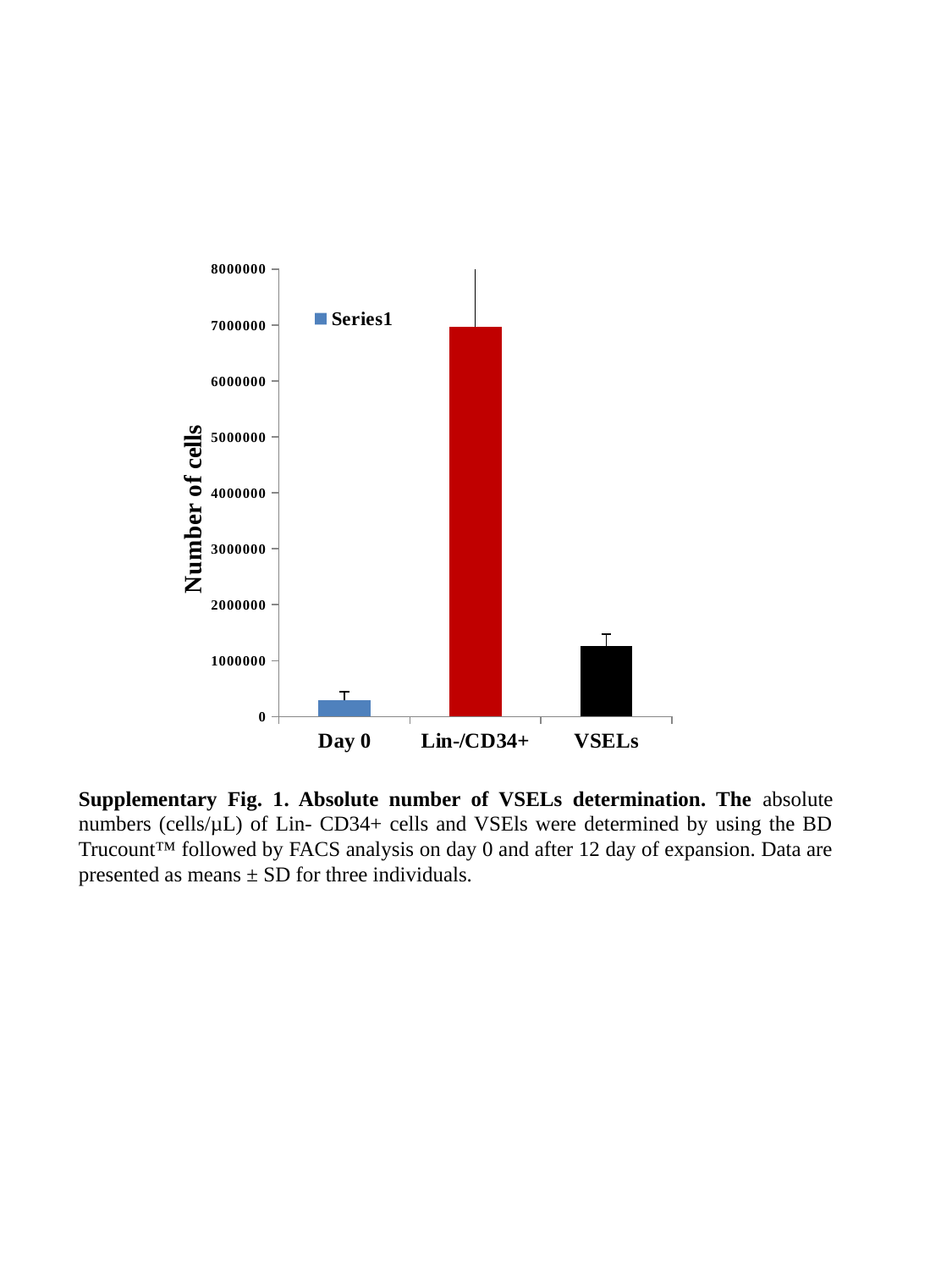

### Chart
| Category | |
|---|---|
| Day 0 | 296666.6666666667 |
| Lin-/CD34+ | 6961103.094436427 |
| VSELs | 1267019.4003527337 |Supplementary Fig. 1. Absolute number of VSELs determination. The absolute numbers (cells/µL) of Lin- CD34+ cells and VSEls were determined by using the BD Trucount™ followed by FACS analysis on day 0 and after 12 day of expansion. Data are presented as means ± SD for three individuals.

## Slide 2
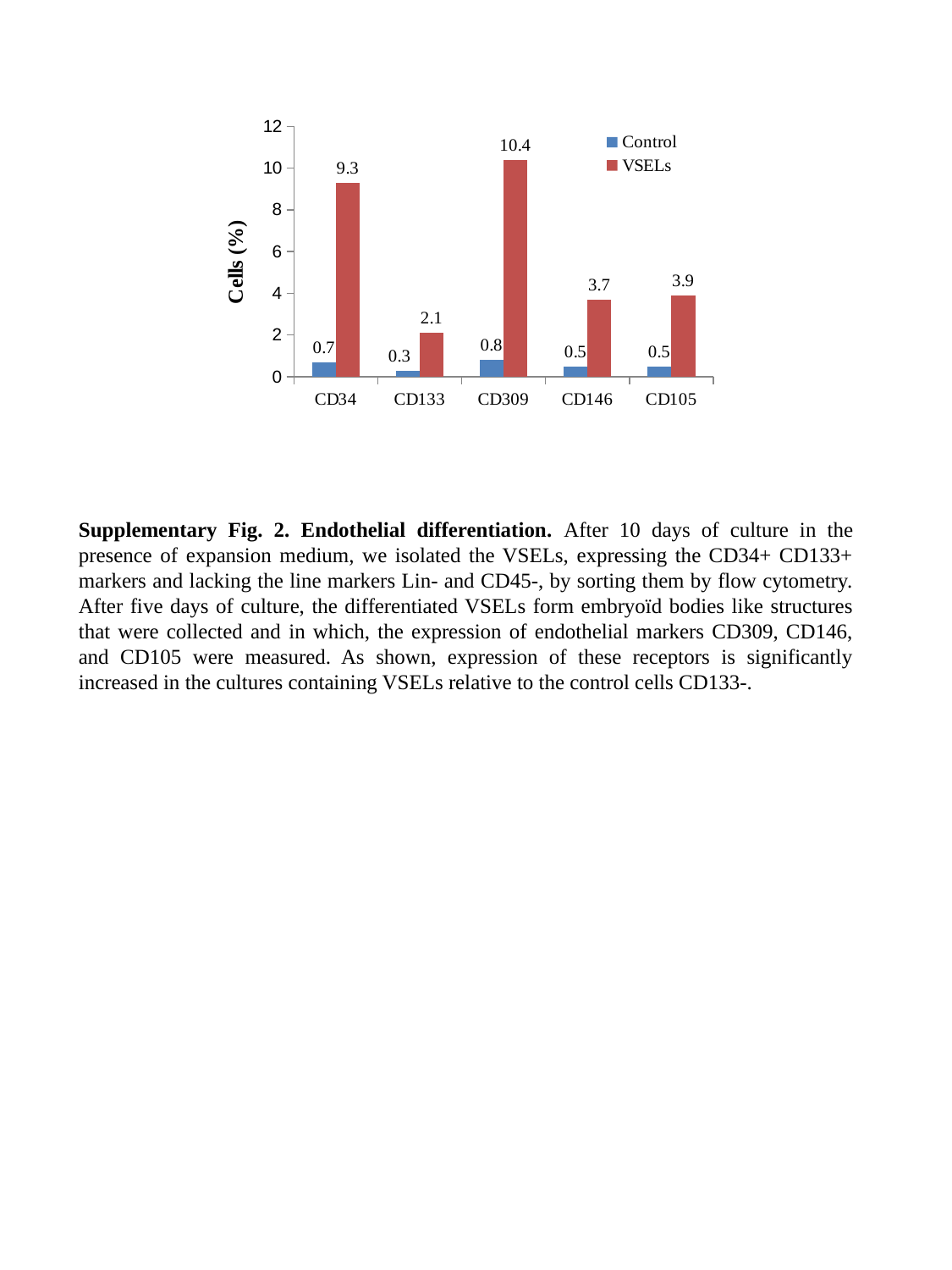

### Chart
| Category | Control | VSELs |
|---|---|---|
| CD34 | 0.7 | 9.3 |
| CD133 | 0.3 | 2.1 |
| CD309 | 0.8 | 10.4 |
| CD146 | 0.5 | 3.7 |
| CD105 | 0.5 | 3.9 |Supplementary Fig. 2. Endothelial differentiation. After 10 days of culture in the presence of expansion medium, we isolated the VSELs, expressing the CD34+ CD133+ markers and lacking the line markers Lin- and CD45-, by sorting them by flow cytometry. After five days of culture, the differentiated VSELs form embryoïd bodies like structures that were collected and in which, the expression of endothelial markers CD309, CD146, and CD105 were measured. As shown, expression of these receptors is significantly increased in the cultures containing VSELs relative to the control cells CD133-.

## Slide 3
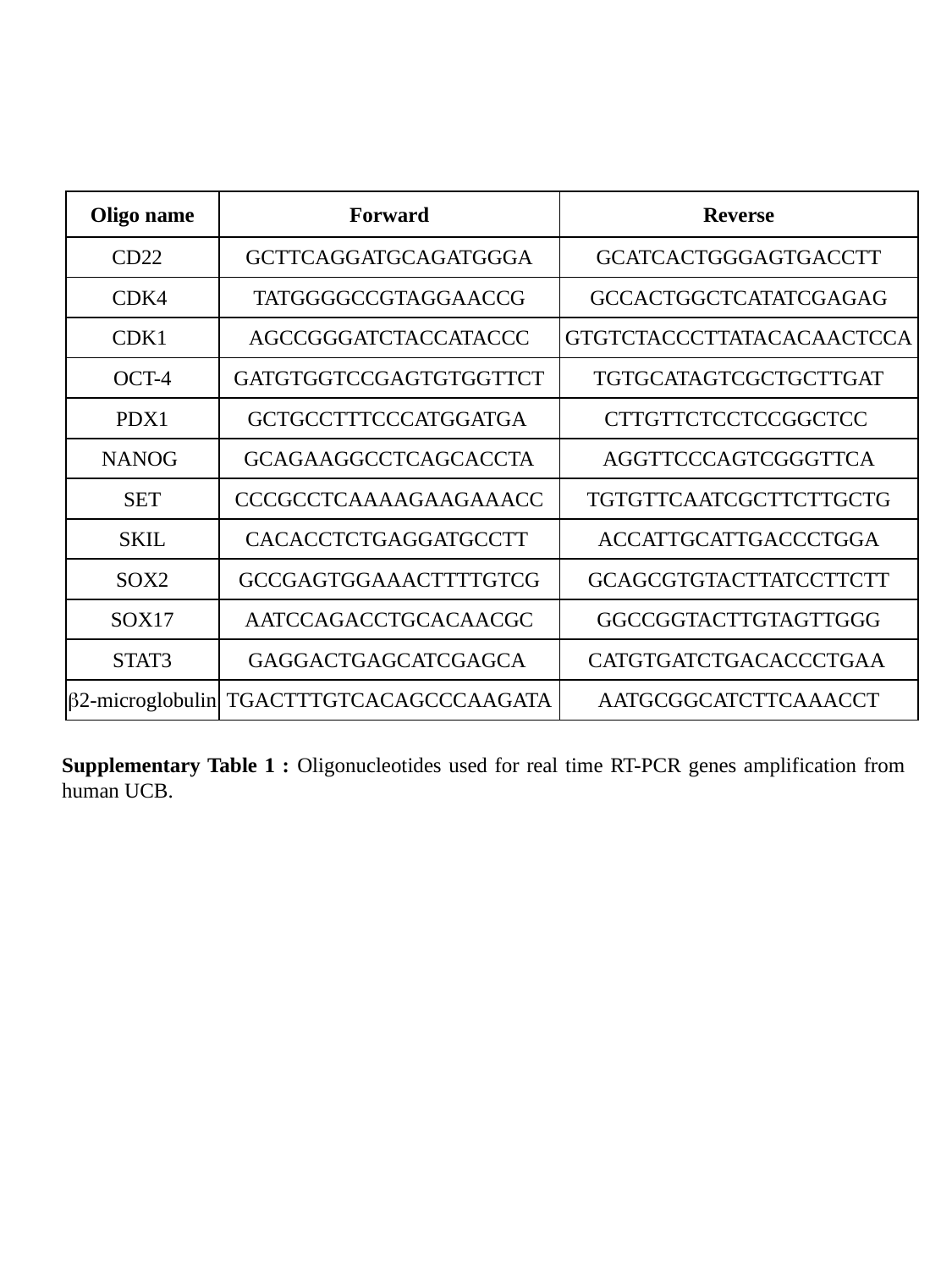

| Oligo name | Forward | Reverse |
| --- | --- | --- |
| CD22 | GCTTCAGGATGCAGATGGGA | GCATCACTGGGAGTGACCTT |
| CDK4 | TATGGGGCCGTAGGAACCG | GCCACTGGCTCATATCGAGAG |
| CDK1 | AGCCGGGATCTACCATACCC | GTGTCTACCCTTATACACAACTCCA |
| OCT-4 | GATGTGGTCCGAGTGTGGTTCT | TGTGCATAGTCGCTGCTTGAT |
| PDX1 | GCTGCCTTTCCCATGGATGA | CTTGTTCTCCTCCGGCTCC |
| NANOG | GCAGAAGGCCTCAGCACCTA | AGGTTCCCAGTCGGGTTCA |
| SET | CCCGCCTCAAAAGAAGAAACC | TGTGTTCAATCGCTTCTTGCTG |
| SKIL | CACACCTCTGAGGATGCCTT | ACCATTGCATTGACCCTGGA |
| SOX2 | GCCGAGTGGAAACTTTTGTCG | GCAGCGTGTACTTATCCTTCTT |
| SOX17 | AATCCAGACCTGCACAACGC | GGCCGGTACTTGTAGTTGGG |
| STAT3 | GAGGACTGAGCATCGAGCA | CATGTGATCTGACACCCTGAA |
| b2-microglobulin | TGACTTTGTCACAGCCCAAGATA | AATGCGGCATCTTCAAACCT |
Supplementary Table 1 : Oligonucleotides used for real time RT-PCR genes amplification from human UCB.
